# Supplementary material for: A Biomimetic Treadmill-Driven Ankle Exoskeleton: A Study in Able-Bodied Individuals
Source: Biomimetics (Basel). 2025 Sep 21;10(9):635. doi: 10.3390/biomimetics10090635 (PMC12468005; doi:10.3390/biomimetics10090635)
Supplement: Supplementary file 1 [file biomimetics-10-00635-s001.zip › biomimetics-3861006-supplementary/Supplementary Files/SupplementaryFile1.pdf]

ID	Age (years)	Height (cm)	Shoe size	Weight (kg)
1	27	183	44	72.5
2	27	181	44	82.2
3	27	185.5	44	98.5
4	27	180	42	75.3
5	28	168.7	42	83.1
6	28	171	42	75.3
7	25	180	44	87.0
8	27	176.5	42	72.5
9	28	182	42	75.5
10	28	179.5	42	89.0
11	27	177.5	42	75.4
12	28	172	44	82.5

Supplementary Table S1: Participant demographics

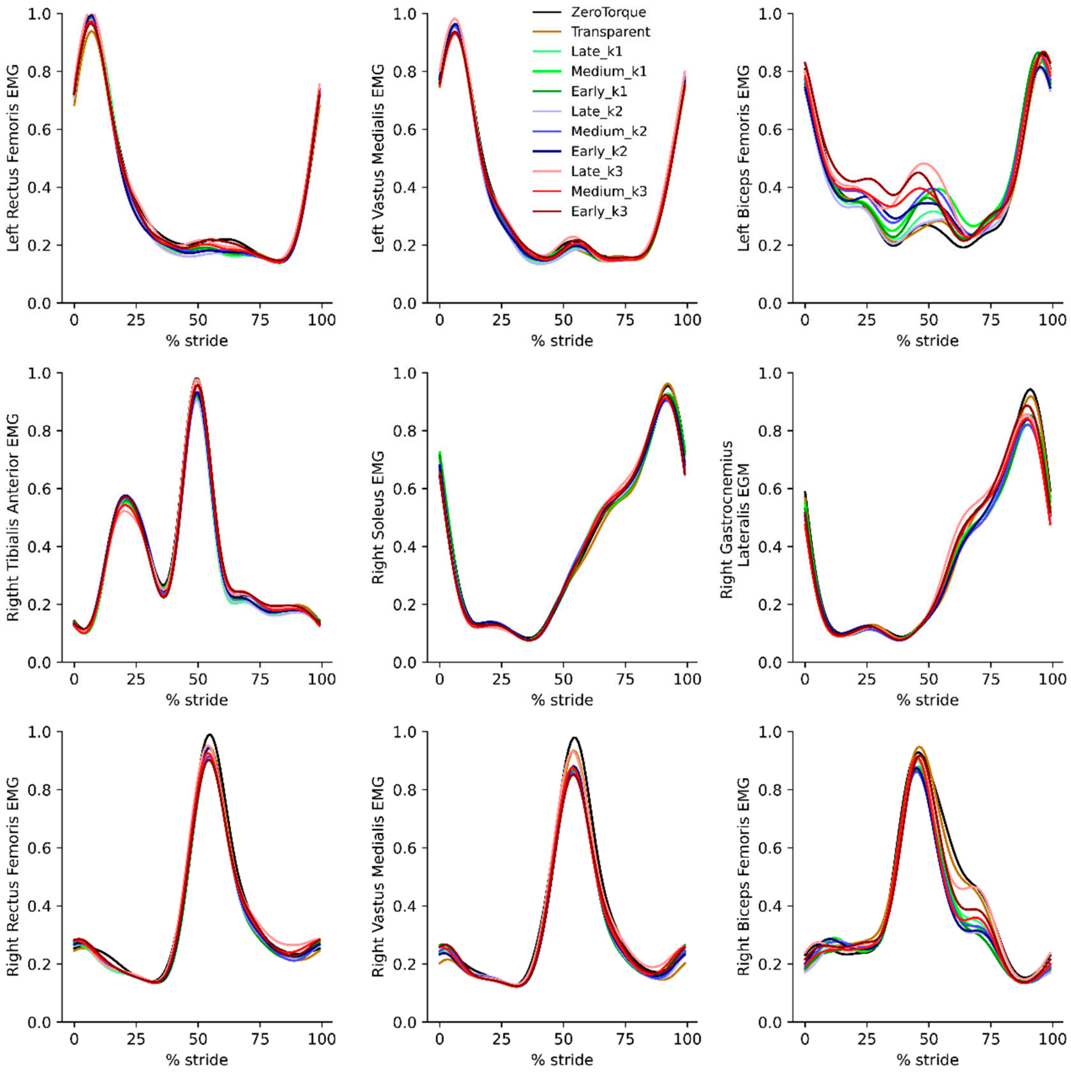

Supplementary Figure S1: EMG results for all measured muscles, omitted from the main document.

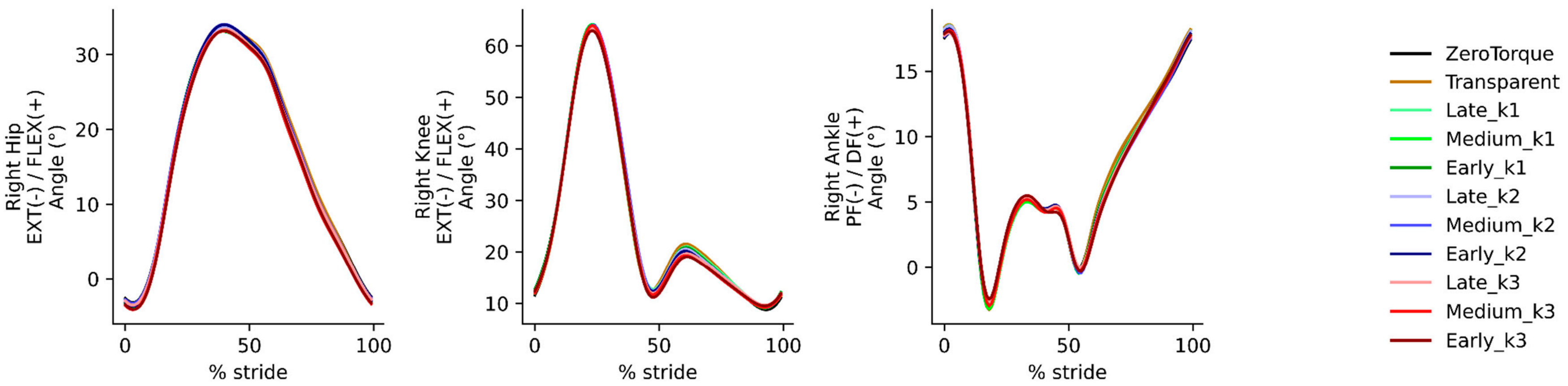

Supplementary Figure S2: Joint kinematics of the contralateral (right) leg. Note that 0% stride is set to left heel strike

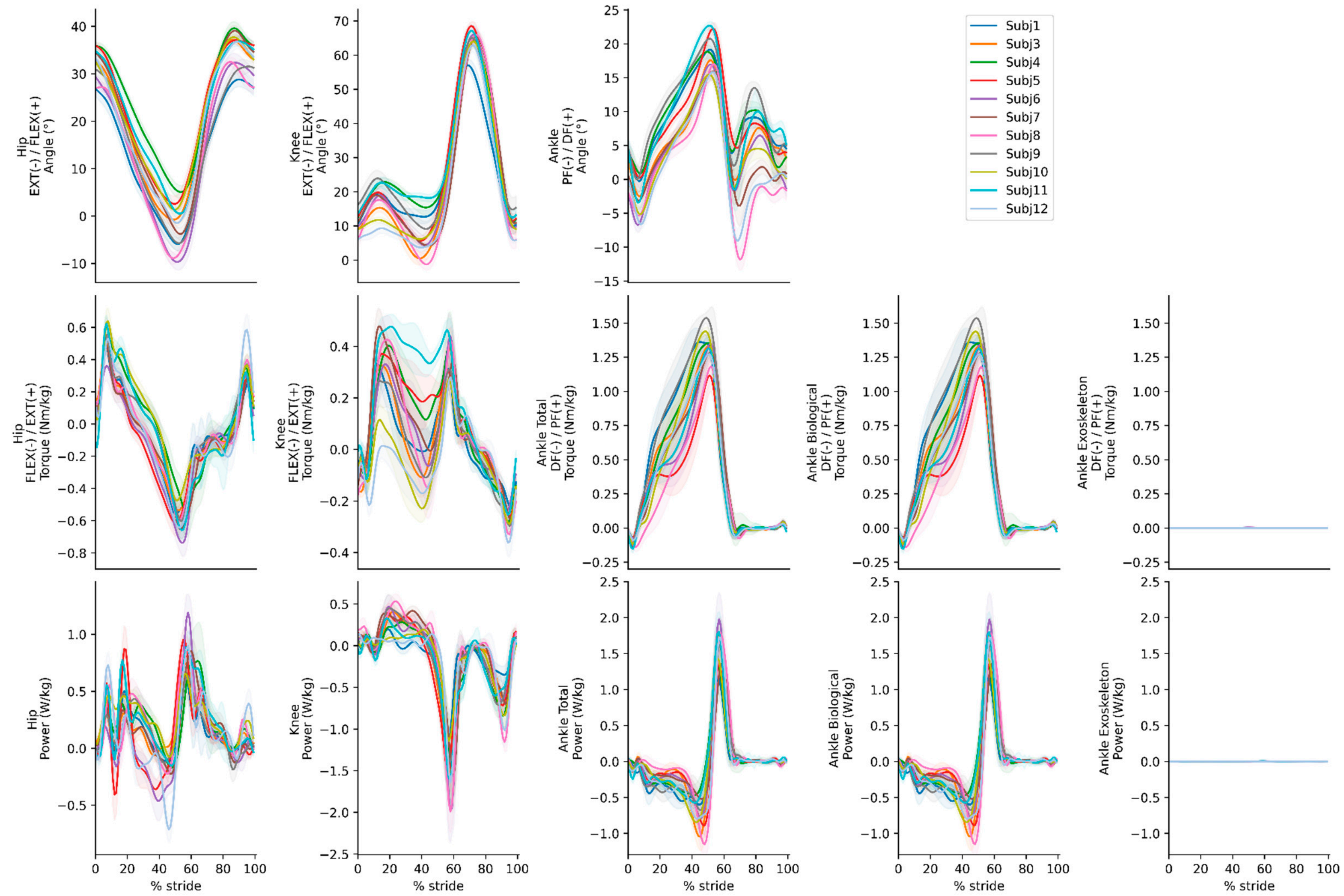

Supplementary Figure S3: Intersubject variability: Biomechanics during ZeroTorque condition

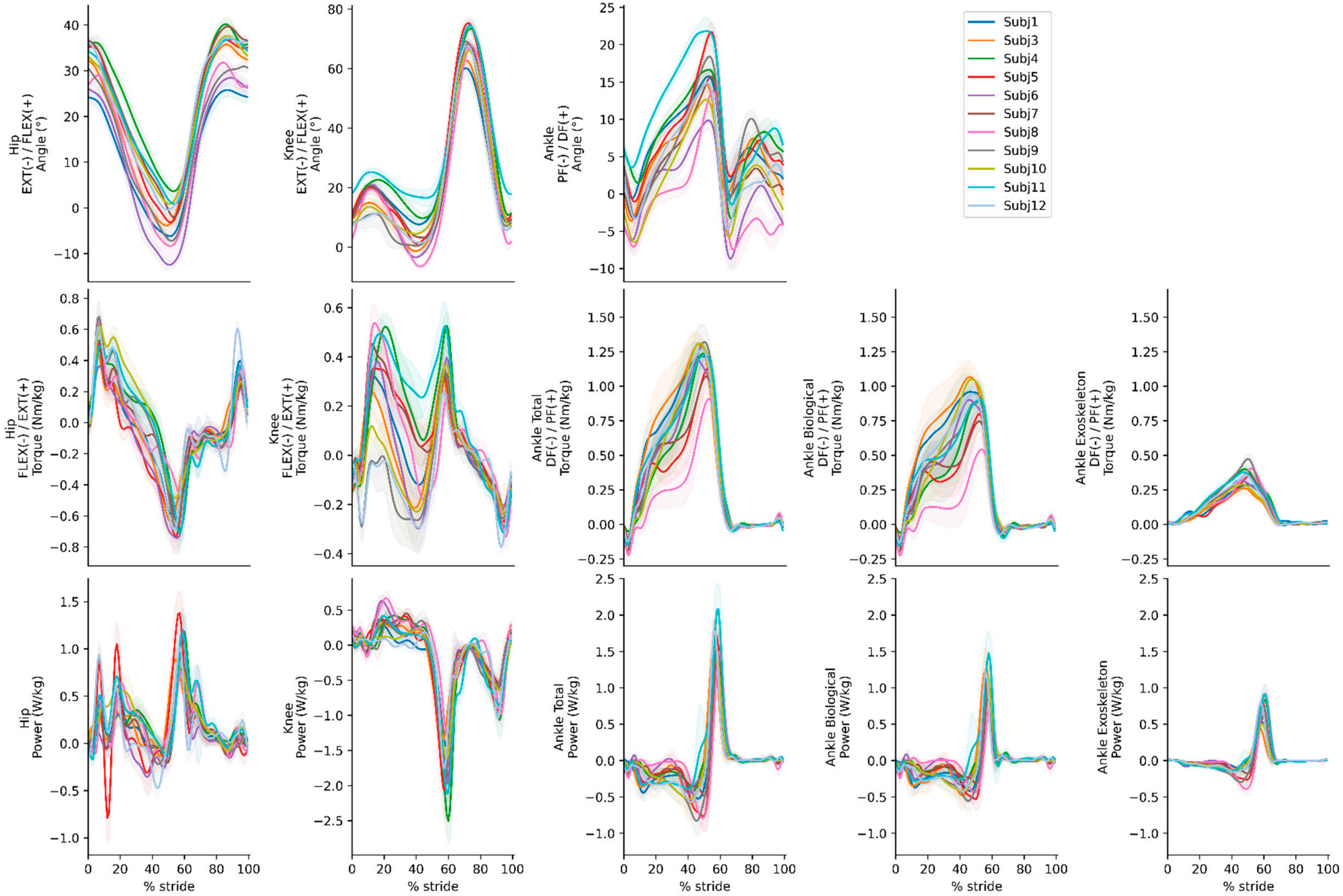

Supplementary Figure S4: Intersubject variability: Biomechanics during Early_k3 condition
